# A protoplast-based transient gene expression assay for the identification of heat and oxidative stress-regulatory genes in perennial ryegrass

**DOI:** 10.1186/s13007-024-01192-5

**Published:** 2024-05-09

**Authors:** Shanshan Lei, Yaolong Zhu, Weiyu Jia, Jing Zhang, Yingjun Chi, Bin Xu

**Affiliations:** College of Agro-Grassland Science, Nanjing Agricultural University, Nanjing, 210095 People’s Republic of China

**Keywords:** Ryegrass, Protoplast, Oxidative stress, Heat stress, Transient gene expression

## Abstract

**Background:**

With the accumulating omics data, an efficient and time-saving transient assay to express target genes is desired. Mesophyll protoplasts, maintaining most stress-physiological responses and cellular activities as intact plants, offer an alternative transient assay to study target genes’ effects on heat and oxidative stress responses.

**Results:**

In this study, a perennial ryegrass (*Lolium* *perenne* L.) mesophyll protoplast-based assay was established to effectively over- or down-regulate target genes. The relative expression levels of the target genes could be quantified using RT-qPCR, and the effects of heat and H_2_O_2_-induced oxidative stress on protoplasts’ viability could be quantitatively measured. The practicality of the assay was demonstrated by identifying the potential thermos-sensor genes *LpTT3.1*/*LpTT3.2* in ryegrass that over-expressing these genes significantly altered protoplasts’ viability rates after heat stress.

**Conclusion:**

This protoplast-based rapid stress regulatory gene identification assay was briefed as ‘PRIDA’ that will complement the stable genetic transformation studies to rapidly identify candidate stress-regulatory genes in perennial ryegrass and other grass species.

## Introduction

With increasingly accumulated omics data, time-saving assays for rapid identification of target genes are highly desirable. To identify and characterize candidate stress-regulatory genes, over-expressing or knocking out/down the target genes is required. Yet, generating stable transgenic plants can be both time-consuming and challenging for most non-model species. Transient assays, such as virus-induced gene silencing (VIGS) [12], *Agrobacterium*-inoculation or Agroinfiltration [3, 6], and protoplast transfection [18], were developed to complement the method of stable genetic transformation to identify or narrow down the list of candidate genes in a short while.

Mesophyll protoplasts are not only amenable to DNA transfection but also maintain most stress-physiological responses and cellular activities as intact plants [13]. Therefore, uniform and abundant isolated protoplasts offer a versatile cell-based experimental system to investigate protein subcellular localization, protein–protein interaction, cell-autonomous regulation, and oxidative stress- and innate immune responses [1, 5, 15, 18].

Perennial ryegrass (*Lolium* *perenne* L.) is an important perennial cool-season grass widely used for turf and forage purposes. Stable genetic transfection of the grass takes at least 7 months from callus initiation to the regeneration of transgenic plants [4]. Ryegrass leaves covered with shining thick cuticles are not amenable to Agroinfiltration or biolistic-based transient gene expression assays. Therefore, a mesophyll protoplast isolation and DNA transfection protocol were developed for protoplast-based transient gene expression assays [19]. This transient assay has been applied in studies on the effects of exogenous supplementation of sodium copper chlorophyllin on H_2_O_2_-induced oxidative stress tolerance [20] and on a target gene’s (*LpCYP72A15*) effect on osmotic and oxidative stress tolerance [15].

In this study, we aimed to use the ryegrass mesophyll protoplast-based transient assay to identify candidate heat and oxidative stress-regulatory genes. Using this assay, we have identified several stress-regulatory genes including the potential thermosensors in ryegrass, *Thermo-tolerance* *3.1* and *3.2* *(LpTT3.1* *&* *LpTT3.2*) [22].

## Materials and methods

### Plant material and growth conditions

Two ryegrass varieties were used in this study, namely cv. ‘Buena vista’ (heat-sensitive variety) and cv. ‘Xialu 6#’ (heat-tolerant variety bred by our group). A healthy plant material is a prerequisite for successful protoplast isolation, transfection, and the protoplast-based gene expression assay. Perennial ryegrass plants were grown in vermiculite: perlite: peat moss (1:3:9) in a growth chamber at 25/20 °C (day/night), 14/10 light/dark photoperiod, watered twice a week, and fertilized once a week with half-strength Hoagland solution. The middle section of the first fully expanded leaf was used as the material for the isolation of protoplasts.

### Gene cloning and vector construction

Perennial ryegrass *NON-YELLOW* *COLOURING* *1-like* *gene* (*LpNOL*) [21], *STAYGREEN* (also named as *NON-YELLOWING* *1*, *LpSGR*) [17], *Thermo-tolerance* *3.1* of *L.* *perenne* (*LpTT3.1*, NCBI Genebank NO.: OR282705) and *LpTT3.2* (Genebank NO.: OR282706) were used in this study. *LpSGR* and *LpNOL* were two characterized genes already known for their roles in plant heat tolerance using stable transgenic ryegrass [8, 20, 23], and the two uncharacterized genes (*LpTT3.1* and *LpTT3.2*) were orthologous to the rice *OsTT3.1* and *OsTT3.2* which were known as potential thermosensors and regulators in rice [22].

To generate the RNAi hairpin structure, the selected fragments of *LpSGR* and *LpNOL* were cloned into the SalI/EcoRI and HindIII/XbaI sites pENTR D-Kannibal vector [16], and then subcloned to the pVT1629 vector [16] driven under the maize ubiquitin promoter using the LR clonase (Invitrogen). To over-express *LpTT3.1* and *LpTT3.2*, we cloned these two genes using the pENTR/D vector first (Invitrogen) and then subcloned them to the pVT1629 vector using the LR clonase as well.

### Protoplast isolation and transfection

The procedure of ryegrass mesophyll protoplast isolation and transfection was described [19]. In brief, the first fully expanded leaves were taken, washed twice, and then blotted dry with absorbent paper. The middle parts of the leaves (~ 0.5 g) were minced into fine fragments about 0.5–1 mm wide in parallel to the leaf vein in a Petri dish containing the enzymatic hydrolysate. Then, the Petri dishes were vacuumed for 30 min using a vacuum pump (60L min^−1^ pumping rate, Product model: GM-1.0A, Jinteng Co., TianJin, China), and then gently rotated on a horizontal shaker for enzyme digestion for 4 h (h) at room temperature. The enzymatic hydrolysate was filtered through a sterilized 75 μm nylon mesh in a 50 ml centrifuge tube with 100 × *g* centrifugation force for 3 min at room temperature. The supernatant was gently removed with a pipette. To each 50 mL centrifuge tube, 20 mL of sterilized W5 solution was added to gently resuspend the protoplasts. Then, the protoplasts were precipitated with centrifugation at 100 × *g* for 3 min, resuspended with 15 mL sterilized MMG solution, and then centrifuged for another 3 min. After removing the supernatant, the precipitated protoplasts were resuspended in 2 ml of MMG solution and kept on ice before transfection. The density of protoplasts (~ 5 × 10^5^ cells per ml) was counted with a hemocytometer under a light microscope (Olympus Model BX53, Tokyo, Japan).

Then 10 μg plasmids and 100 μl protoplasts were added into 1.5 ml centrifuge tubes (the tubes were already rinsed with 1% BSA solution) and gently mixed; then 110 μl of pre-warmed PEG4000 solution (42 °C) was added to each centrifuge tube, mixed gently to a homogeneous liquid phase, and stood at room temperature for 20 min. Then, 800 μl of W5 solution was slowly added along the tube wall to each centrifuge tube, gently mixed to a homogeneous liquid phase, centrifuged at 100 × *g* for 3 min, and resuspended with 800 μl W5 solution. The protoplast integrity was detected again under a light microscope. The transfected protoplasts were placed in a 25 °C incubator for 16 h to allow the transgenes’ expression [19].

### Reagents and solutions

The formula and preparation procedures of reagents and solutions are listed in Table 1 according to Yu et al. [19].

**Table 1:** Solutions for ryegrass mesophyll protoplast isolation and transfection

Solutions	Components	Note
Enzymatic hydrolysate	0.6 M D-Mannitol	Dissolve in dH_2_O with a final volume of 20 mL and filter through the 75 μm nylon mesh. Freshly prepared before use
	10 mM MES·H_2_O	
	0.3 g Cellulase Onozuka R-10	
	0.15 g Macerozyme R-10	
W5 solution	154 mM NaCl	Dissolve in dH_2_O with a final volume of 200 mL and filter through the 75 μm nylon mesh. Freshly prepared before use
	2 mM MES·H_2_O	
	125 mM CaCl_2_	
	5 mM KCl	
MMG solution	0.6 M D-Mannitol	Dissolve in dH_2_O with a final volume of 20 mL and filter through the 75 μm nylon mesh. Freshly prepared before use
	4 mM MES·H_2_O	
	15 mM MgCl_2_	

### Heat stress and H_2_O_2_ treatments of transfected protoplasts

After 16 h of incubation, the transfected protoplasts were ready for heat and oxidative stress treatments. For heat stress, tubes containing protoplasts were transferred to 35 ℃ or at 31 °C, 33 °C, 35 °C, 37 °C, 39 °C, or 41 °C for temperature range test, in water baths for 20 min, stained with 1 μl of 0.1% Evans blue dye for 1 min and then observed under microscope (× 100). Three representative optical fields were photographed.

For H_2_O_2_-induced oxidative stress, 30% H_2_O_2_ solution was added to the suspended protoplasts in a centrifuge tube to make the final H_2_O_2_ concentration of 25, 37.5, or 50 mM, incubating for 5 min at room temperature (note: To make 1000 mM H_2_O_2_ stock solution, add 10.21 ml 30% H_2_O_2_ solution to 89.79 ml water. The H_2_O_2_ solution shall be freshly made before use). Then, 1 μL of 0.1% Evan blue solution was added and gently mixed, incubating for 1 min, and then immediately photographed at the microscope as described above.

Broken protoplasts stained in blue were counted as dead protoplasts and the number of viable and total protoplasts was counted. Protoplast viability (%) was counted as the number of intact, unstained protoplasts divided by the number of total protoplasts.

For the detection of relative expression levels of *LpTT3.1* and *LpTT3.2* in ryegrass after heat stress, potted ryegrass plants (cv. ‘Buena vista’ and cv. ‘Xialu 6#’) were treated under 38 °C for 24 h (h) in a growth chamber. The fully expanded leaves were harvested from the plants for RNA extraction.

### RNA extraction and RT-qPCR

The MolPure® cell RNA kit (Yeasen Biotech. Co., Ltd., Shanghai, China) was used for RNA extraction from protoplasts, and the MolPure® Plant RNA Kit (Yeasen Biotech. Co) was used for RNA extraction from the whole leaves. The quantity and integrity of total RNA were checked using Nanoready (NanoDrop Technologies, Hangzhou, China) and by running through a 1% agarose gel. The cDNA synthesis was performed using the ChamQ Universal SYBR qPCR Master Mix for RT-PCR Kit (Vazyme Biotech Co., Nanjing, China) with oligo-dT primers. For reverse transcription-quantitative polymerase chain reaction (RT-qPCR), PRIMER EXPRESS_ software (v3.0; Applied Biosystems, Foster City, CA, USA) was used to design primer sets for *LpTT3.1*, *LpTT3.2*, *LpSGR*, *LpNOL* and the reference gene (*LpeIF4A*) [7] (Table 2). Each sample had three replicates, and the data were normalized against the reference gene *LpeIF4A*.

**Table 2:** Primers used in the study

Primer name	Sequence (5′–3′)
*LpeIF4A*-qRT F	AACTCAACTTGAAGTGTTGGAGTG
*LpeIF4A*-qRT R	AGATCTGGTCCTGGAAAGAATATG
*LpTT3*.*1*-qRT F	ATGCAGCGGCGGCGGG
*LpTT3*.*1*-qRT R	AACATCATATACGGGCATGCGTTC
*LpTT3*.*2*-qRT F *LpTT3*.*2*-qRT R *LpSGR*_qRT F *LpSGR*_qRT R *LpNOL*_qRT F *LpNOL*_qRT R	ATGGCTTCCTCCATCGC CAATGTTTTATCCTTCCTCTCTA GAGGAGGCGAACTCGAAG GGTTGTACCACCCTTGCAG GCTGGCAAAGAAGTTTCTCA ATGCTGCTCTCCAAATTCCT

### Statistical analysis

Data were statistically analyzed by One-way ANOVA at a significance level of 0.05 using SPSS20.0 software (SPSS Inc.). Data are expressed as means ± standard error. At least two separate experiments were carried out for each test.

## Results

### Suppression of *LpNOL* or *LpSGR* improved or compromised heat and oxidative stress tolerance in mesophyll protoplasts

**Fig. 1:**
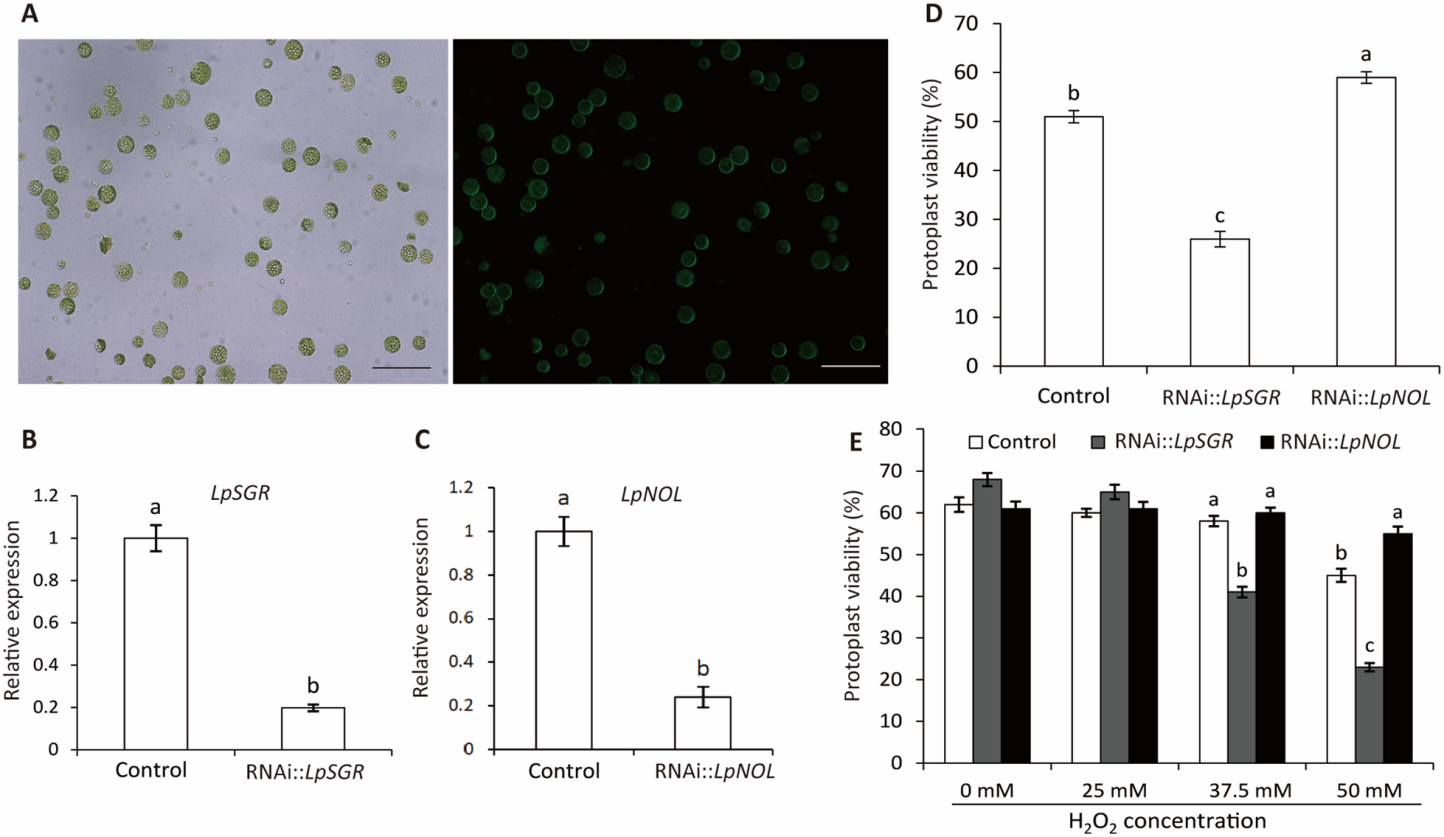
Transient suppression of *LpNOL* or *LpSGR* altered heat and oxidative stress tolerances of ryegrass protoplasts (cv. ‘Buena Vista’). **A** Ryegrass protoplasts transfected with pVT1629-GFP visualized under bright (left) and GFP (right) fields. Bars in (**A**) indicate 1 mm. **B**, **C** Relative expressions of *LpSGR* and *LpNOL* after 16 h of protoplasts transfection with the empty vector control and RNAi vectors. **D** Protoplasts’ viability after heat stress at 35 ℃ for 20 min. **E** Protoplasts’ viability under 25–50 mM H_2_O_2_-induced oxidative stress. Noting that the control in (**B**–**E**) was those transfected with empty vectors. At least 30 protoplasts were counted in one optical field, and the means were from 3 optical fields. Different letters represent statistically significant differences at *P* = 0.05, and the bars above the columns represent standard error**s**

More than 90% of the mesophyll protoplasts were successfully transfected with plasmids expressing the GFP reporter gene (Fig. 1A). To test whether the transfected protoplasts was a reliable and sensitive system for characterizing heat-stress regulatory genes, we transfected ryegrass (cv. ‘Buena Vista’) protoplasts with the RNAi vectors to suppress *NON-YELLOW* *COLOURING* *1-like* (*LpNOL*) and *STAYGREEN* (*LpSGR*), respectively. Stable transgenic ryegrass with suppressed *LpNOL* or *LpSGR* have significantly improved or compromised heat tolerance [8, 20, 23]. After DNA transfection, the protoplasts were rested in 25 °C for 16 h, and the relative expression levels of *LpNOL* and *LpSGR* were ~ 20% that of the control ones (Fig. 1B, C). After the resting stage, the protoplasts were subjected to heat treatment (35 °C) for 20 min, then stained with Evans blue for 1 min and immediately observed under the microscope. Consistent with the results of stable transgenic ryegrass plants, transient suppression of *LpNOL* led to 16% higher protoplasts viability, while suppression of *LpSGR* led to 49% lower protoplasts viability than the empty vector control (Fig. 1D).

Oxidative stress is often associated with heat stress on plants with the burst and accumulation of reactive oxygen species (ROS) aggravating cellular damage [9]. Constitutive suppression of *LpNOL* and *LpSGR* also affected the ROS accumulation and the ROS-scavenging system in stable transgenic plants [8, 20, 23]. To test whether this protoplast-based transient assay is also sensitive to oxidative stress treatment, we treated the transfected protoplasts with different concentrations of H_2_O_2_. As shown in Fig. 1E, with H_2_O_2_ at final concentrations of 37.5 to 50 mM, transient suppression of *LpSGR* significantly decreased protoplast viability, while the contrary was true for *LpNOL* (Fig. 1E).

### Identification of potential thermosensors, *LpTT3.1* and *LpTT3.2*, in ryegrass

**Fig. 2:**
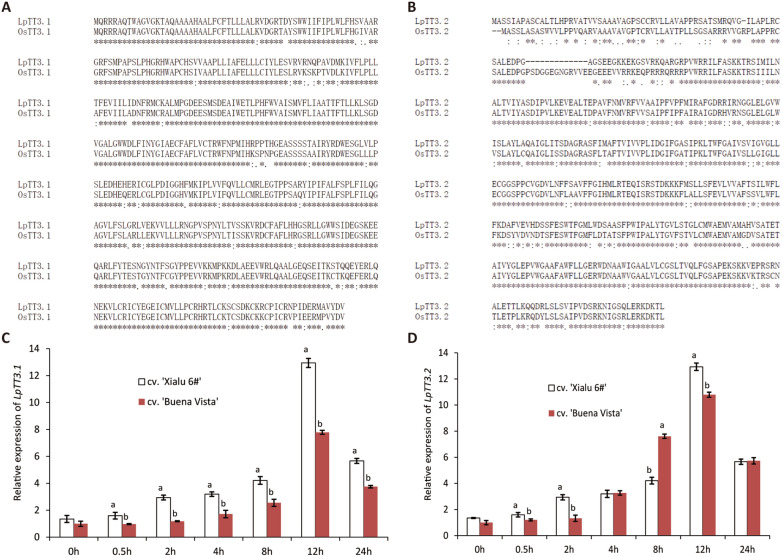
Sequence alignments and heat-responsive expression of *LpTT3.1* & *LpTT3.2*. **A**, **B** Sequence alignment of LpTT3.1 vs OsTT3.1 and LpTT3.2 vs OsTT3.2; **C**, **D** Relative expression of *LpTT3.1* & *LpTT3.2* after heat stress at 38 ℃ in two varieties of perennial ryegrass using RT-qPCR. Different letters represent statistically significant differences at *P* = 0.05, and the bars above the columns represent standard error**s**

To demonstrate this mesophyll-based transient expression system is suitable for identifying candidate stress-regulatory genes, *LpTT3.1* and *LpTT3.2* were chosen as candidate genes for characterization. These two ryegrass genes were orthologous to the rice *TT3.1* and *TT3.2* sharing 86.4% and 74.4% nucleotide (nt) identities, respectively (Fig. 2A, B). Expression levels of both *LpTT3.1* and *LpTT3.2* increased after 2 h of heat stress, the expression of *LpTT3.1* was more sharply increased in the heat-tolerant variety (cv. ‘Xialu 6#’) than in the sensitive one (cv. ‘Buena Vista’) (Fig. 2C, D).

**Fig. 3:**
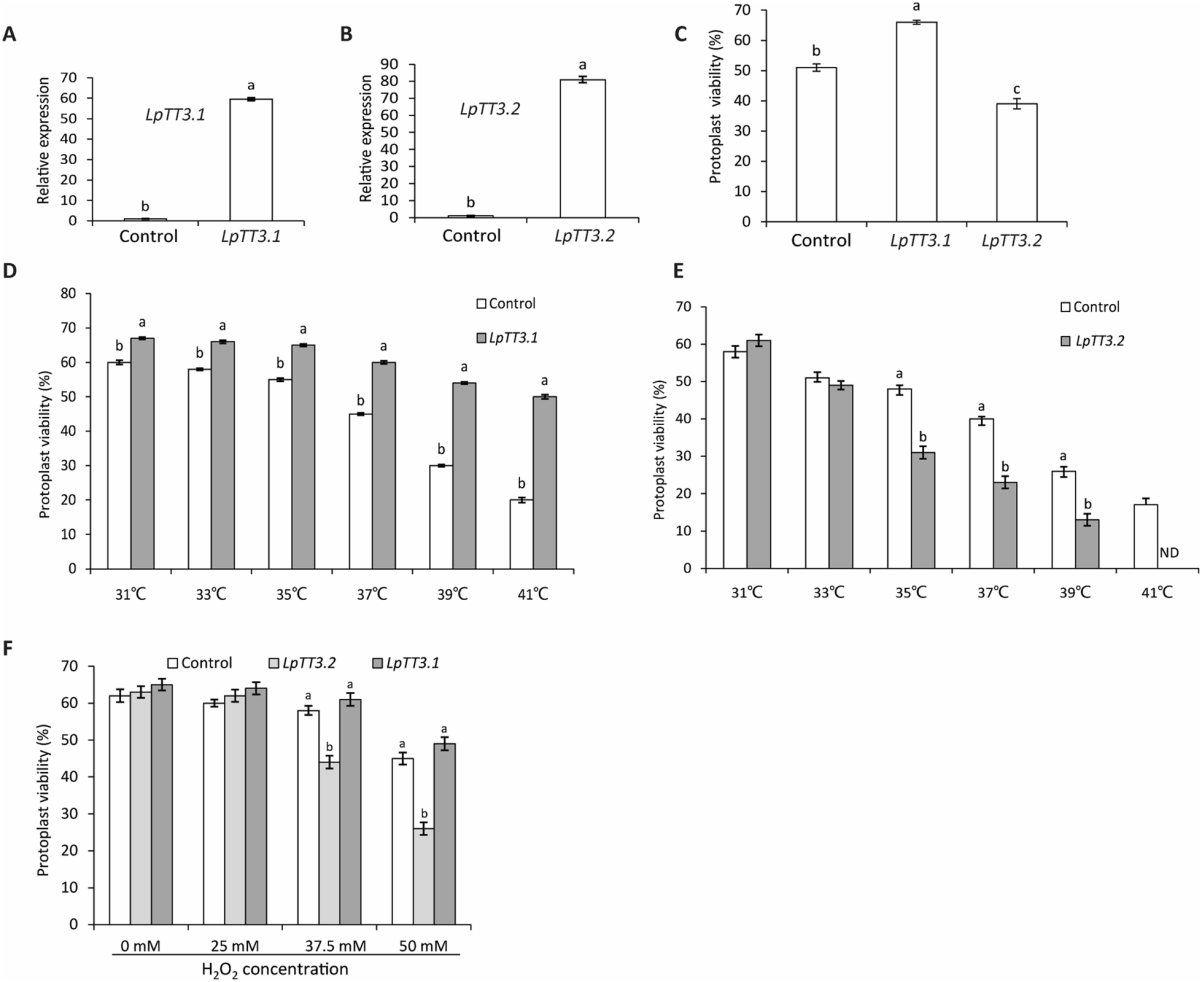
Transient over-expression of *LpTT3.1* or *LpTT3.2* altered heat and oxidative stress tolerances of ryegrass protoplasts (cv. ‘Buena Vista’). **A**, **B** Relative expression levels of *LpTT3.1* and *LpTT3.2* after 16 h of protoplast transfection with the empty vector control or target genes under a *ubiquitin* promoter; **C** Protoplast viability after heat stress at 35 ℃ for 20 min; **E**, **F** Protoplasts’ viabilities under heat stress at 31–41℃ for 20 min or under 25–50 mM H_2_O_2_-induced oxidative stress. Noting that the control in (**C**–**F**) was those transfected with empty vectors. ‘ND’ in (**G**) is the abbreviation for ‘not detected’. At least 30 protoplasts were counted in one optical field, and the means were from 3 optical fields. Different letters represent statistically significant differences at *P* = 0.05, and the bars above the columns represent standard error**s**

Using the protoplasts of cv. ‘Buena Vista’, transient over-expressing *LpTT3.1* resulted in 58 times higher expression levels and led to 29% higher protoplast viability rate after heat stress treatment than those of the control (Fig. 3A, C); while transient over-expressing *LpTT3.2* resulted in 82 times higher expression levels but 24% less protoplast viability rate after heat stress (Fig. 3B, C). To optimize the temperature range for heat stress, we treated protoplast over-expressing *LpTT3.1* & *LpTT3.2* at 31 to 41 °C, and the result showed that 35 to 39 ℃ was the optimal treatment condition (Fig. 3D, E). Furthermore, over-expressing *LpTT3.2* significantly decreased protoplast viability when treated with 25 to 50 mM H_2_O_2_, while over-expressing *LpTT3.1* did not affect protoplast viability under oxidative stress (Fig. 3F).

**Fig. 4:**
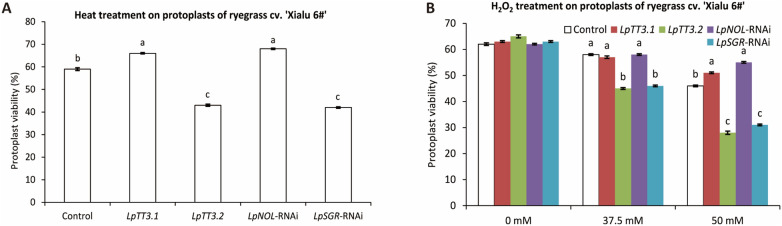
Transient gene suppression/over-expression of *LpNOL*, *LpSGR*, *LpTT3.1*, or *LpTT3.2* altered heat and oxidative stress tolerances of ryegrass protoplasts (cv. ‘Xialu 6#’). Protoplasts’ viabilities under heat stress at 37℃ for 20 min (**A**) or under H_2_O_2_-induced oxidative stress (**B**). Noting that the control was those transfected with empty vectors. At least 30 protoplasts were counted in one optical field, and the means were from 3 optical fields. Different letters represent statistically significant differences at *P* = 0.05, and the bars above the columns represent standard error**s**

To verify whether the above-selected stress conditions were effective for other ryegrass varieties, we tested it in a more heat-tolerant variety (cv. ‘Xialu 6#’). As shown in Fig. 4, the viabilities of transfected protoplasts after heat treatment at 37 °C or after 37.5 to 50 mM H_2_O_2_ treatment differed according to the target genes, and the results were consistent with those obtained with ryegrass cv. ‘Buena Vista’.

## Discussion

To explore the post-genomics data, time-saving and high throughput functional genomic assays are indispensable to identify target genes. After the release of perennial ryegrass genome [2, 10], multiple comparative transcriptome analyses and Genome-Wide Association Studies (GWAS) were carried out [11, 14, 24], providing numerous candidate genes for further functional studies. Generation of stable transgenic ryegrass plants takes at least 7 months, and more time is needed to characterize these transgenic plants for heat and/or oxidative tolerance. To complement the extremely time-consuming stable genetic transformation procedure, here we report an alternative assay using the mesophyll protoplast-based transient gene expression system for rapid identification of stress regulatory genes in ryegrass. Therefore, we named this protocol as ‘PRIDA’ for Protoplast-based Rapid stress regulatory gene Identification Assay. Compared with ectopic expression in yeast or in *Arabidopsis*, the PRIDA assay is carried out in the native plant species and takes even less time.

Leaves of perennial ryegrass are narrow (3–5 mm width) with parallel veins and shining cuticles that are recalcitrant to *Agrobacterium*- or biolistic-based transient gene expression assays. Yet, abundant mesophyll protoplasts can be isolated from its green leaves, the protoplast DNA transfection rate can be higher than 90%, and these freshly isolated protoplasts maintain most of their innate cellular activities and are sensitive to abiotic stress conditions [1, 5, 15, 18]. All these features make it a feasible system to characterize the target gene’s regulatory roles in stress. In a recently reported study, we used this PRIDA assay to analyze the target gene’s (*LpCYP72A15*) effect on osmotic/dehydration stress [15]. In this study, we further showed that this PRIDA assay, capable of both over-expressing and suppressing the target gene, was an effective way to identify genes involved in heat and oxidative stress. A similar assay can be developed in other non-model grass species as well for the identification and characterization of genes regulating heat, oxidative, osmotic, and other types of abiotic stresses.

Fine-tuning the stress treatment conditions is a key to the successful interpretation of the results of PRIDA. Perennial ryegrass is a cool-season grass species that is sensitive to heat stress [24]. Yet, treating ryegrass mesophyll protoplasts below 33 ℃ cannot tell the stress difference in a short time, while treatment at 41 ℃ for 20 min made the protoplasts stick to each other, making it impossible to count precisely. Therefore, the optional temperature range for heat treatment is 35 to 39 ℃. Treatment with 100 mM H_2_O_2_ killed all protoplasts within 5 min, while treatment with 25 mM H_2_O_2_ could not discriminate the transgenes’ effects. H_2_O_2_ treatment at 37.5–50 mM is the optimal condition. Similarly, mannitol at 100 mM was the optimal condition for osmotic stress treatment in this ryegrass protoplast transient assay [15]. We tested the protocol in two ryegrass varieties with contrasting heat tolerance and the results indicated that the above-selected stress conditions were optimal for both varieties. Yet, when the ‘PRIDA’ system is applied to other grass species, these treatment conditions shall be optimized accordingly.

The *TT3.1* and *TT3.2* genes act as thermosensors in rice that, upon heat stress, both the plasma membrane-localized E3 ligase TT3.1 and the chloroplast-localized TT3.2 proteins translocate to endosomes where TT3.1 trigger the degradation of TT3.2 to protect chloroplast from heat-induced damage [22]. Over-expressing *TT3.1* or *TT3.2* in stable transgenic rice improved or compromised plant heat tolerance, respectively [22]. In this study, using this PRIDA assay, we were able to pin down that this pair of ryegrass genes (*LpTT3.1* & *LpTT3.2*) that likely share the same functions in heat tolerance and can be used as gene editing targets for further molecular breeding of perennial ryegrass.

It should also be noted that these mesophyll protoplasts lack integral cell wall and cannot be kept for more than a week. Therefore, this transient assay cannot be used to study target genes involving cell wall and/or inter-cellular stress-related pathways or their long-term effects on cell division, growth, and development. An in-silico analysis of the target gene/protein (e.g. prediction of target proteins’ subcellular localization) shall be performed before the test.

## Conclusion

**Fig. 5:**
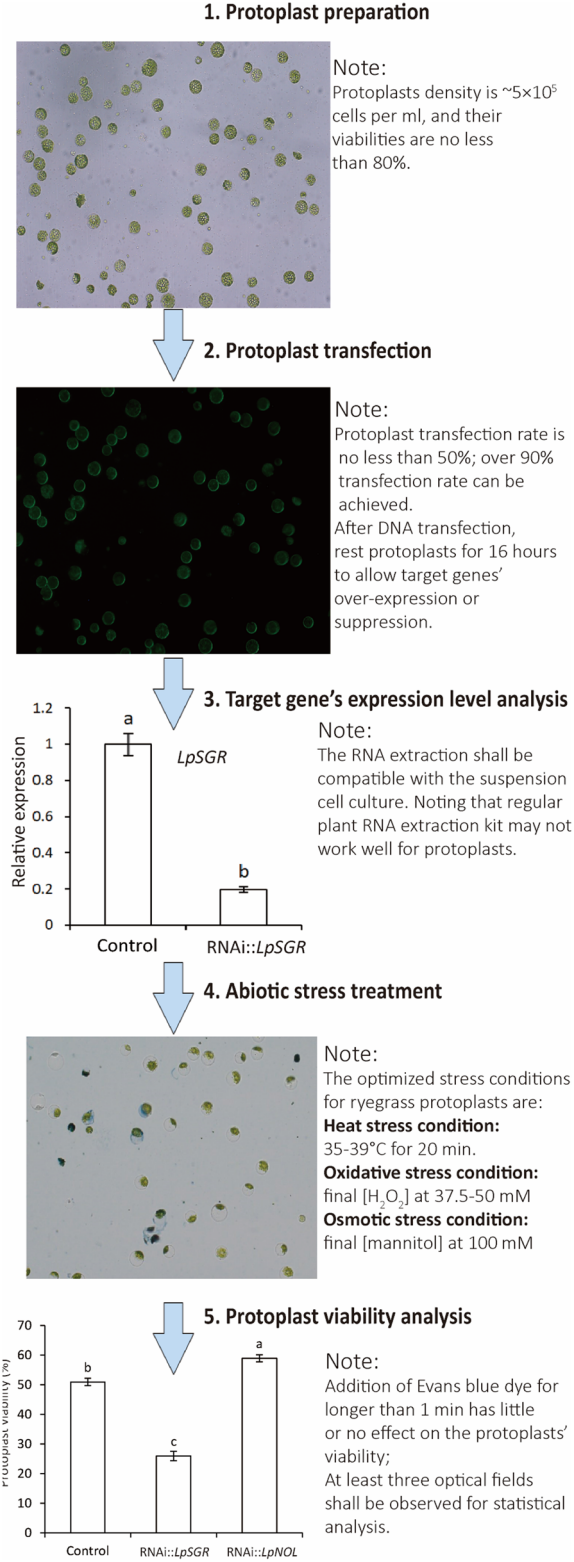
Illustration of the procedure of PRIDA

A detailed procedure of PRIDA was summarized and illustrated in Fig. 5 with critical points pinpointed. The established PRIDA assay in ryegrass can be readily adapted to other types of abiotic stresses and in other plant species to rapidly identify candidate stress-regulating genes.

## Data Availability

The datasets supporting the conclusions and description of a complete protocol are included within the article. The pVT1629 vector is available upon request.
